
JOURNAL INFORMATION
==============================
NLM Title Abbreviation: Buffalo Med J
NLM Journal ID: 0027534

Buffalo Medical Journal

ISSN: 1040-3817
Publisher: Biomedical Journal Digitization Project

ARTICLE INFORMATION
==============================
PMCID: PMC8735500
PMID: 36886912
Article version: 1
Subjects: Item

Item

Print publication date: 1905 Aug
Volume: 61
Issue: 1
First page: 72
Last page: 72
License: This work is free of known copyright restrictions. For more information, please see PMC Back Issue Digitization.
License URL: https://creativecommons.org/publicdomain/mark/1.0/

==============================

A Manual of Acute Poisoning. With Methods for Use in First Aid to the Injured. By John W. Wainwright, M.D. New York: E. R. Pelton. 1905. (Price, 75 cents.)

A Practical Treatise on Fractures and Dislocations. By Lewis A. Stimson, B.A., M.D., Professor of Surgery in Cornell University Medical College, New York; Surgeon to the New York and Hudson Street Hospitals. Fourth edition, thoroughly revised. Octavo, 844 pages with 331 engravings and 46 plates. Philadelphia and New York: Lea Brothers & Co. 1905. (Cloth, $5.00; leather, $6.00; half morocco, $6.50, net prices.)

Saunders's Pocket Medical Formulary. By William M. Powell, M.D., author of "Essentials of Diseases of Children." Containing 1831 formulas from the best known authorities. With an Appendix containing Posological Table, Formulas and Doses for Hypodermic Medication, Poisons and their Antidotes, etc., etc. Seventh edition, revised. Philadelphia and London: W. B. Saunders & Co. 1905. (In flexible morocco, with side index, wallet, and flap, $1.75 net.)

Lea's Series of Medical Epitomes. Edited by Victor C. Pedersen, M.D. Clinical Diagnosis and Uranalysis. A Manual for Students and Practitioners. By James R. Arneill, A.B., M.D., Professor of Medicine and Clinical Medicine in the University of Colorado, Denver. Duodecimo, 244 pages, with 79 engravings and a colored plate. Philadelphia and New York: Lea Brothers & Co. 1905. ($1.00 net.)

### LITERARY NOTE.

Cunningham's Anatomy.—Messrs. William Wood & Company announce a forthcoming new edition of Cunningham's Textbook of Anatomy. During the two years of this book's existence, it has sprung into universal favor and is now the standard text-book in a majority of the prominent medical schools of this country.

### ITEM.

Japanese Post Cards in Battle.—A collector of post cards in Saint Petersburg states, according to the *Philadelphia Public Ledger,* that all the soldiers in the Japanese army are supplied with peculiar post cards. These cards are surrounded with mourning border, printed on a piece of white silk, and are worn by the Japanese soldiers on their chests. Before going to the war they write on the post card the name and address of the person to whom they wish the information in case of death to be sent. In case of death on the field of battle the post cards are stamped with the seal of the regiment, certifying the death of the bearer, and are sent to Japan.

FOR SALE—I will sell my eye and ear practice to a registered physician or optician and guarantee $250.00 a month profit on refraction alone. $1000.00 cash necessary. Dr. J. T. S., 302-517 S. Broadway, Los Angeles, Cal.
